# Electrochemical Migration Behavior of Copper-Clad Laminate and Electroless Nickel/Immersion Gold Printed Circuit Boards under Thin Electrolyte Layers

**DOI:** 10.3390/ma10020137

**Published:** 2017-02-08

**Authors:** Pan Yi, Kui Xiao, Kangkang Ding, Chaofang Dong, Xiaogang Li

**Affiliations:** 1 Corrosion and Protection Center, University of Science and Technology Beijing, Beijing 100083, China; B20160547@xs.ustb.edu.cn (P.Y.); cfdong@ustb.edu.cn (C.D.); lixiaogang99@263.net (X.L.); 2 State Key Laboratory for Marine Corrosion and Protection, Luoyang Ship Material Research Institute, Qingdao 266101, China; dingkk@sunrui.net; 3 Ningbo Institute of Material Technology & Engineering, Chinese Academy of Sciences, Ningbo 315201, China

**Keywords:** electrochemical migration, copper dendrites, PCB-Cu, PCB-ENIG, thin electrolyte layer

## Abstract

The electrochemical migration (ECM) behavior of copper-clad laminate (PCB-Cu) and electroless nickel/immersion gold printed circuit boards (PCB-ENIG) under thin electrolyte layers of different thicknesses containing 0.1 M Na_2_SO_4_ was studied. Results showed that, under the bias voltage of 12 V, the reverse migration of ions occurred. For PCB-Cu, both copper dendrites and sulfate precipitates were found on the surface of FR-4 (board material) between two plates. Moreover, the Cu dendrite was produced between the two plates and migrated toward cathode. Compared to PCB-Cu, PCB-ENIG exhibited a higher tendency of ECM failure and suffered from seriously short circuit failure under high relative humidity (RH) environment. SKP results demonstrated that surface potentials of the anode plates were greater than those of the cathode plates, and those potentials of the two plates exhibited a descending trend as the RH increased. At the end of the paper, an electrochemical migration corrosion failure model of PCB was proposed.

## 1. Introduction

As the swift development of electronic technology, electronic devices or products are moving towards further integration and miniaturization. Unfortunately, the corrosion failure problems of printed circuit boards (PCBs) have been occurring more frequently, and even a small amount of corrosion products can severely affect the reliability of PCBs [1]. Under practical application conditions, the adsorption electrolyte layer may covers on PCBs due to the variation in diurnal temperature. Therefore, a thin electrolyte layer that may contain corrosive particles, whose thickness dynamically varies in the service environment, can cover the surface of PCBs, leading to electrochemical corrosion failure in a dynamic thin electrolyte layer.

As we know, Cu and its alloys always exhibit superior performance due to its exceptional electrical and thermal conductivity, cut-price, excellent reliability of solder joints, etc. [2,3], so they are often used as the substrate materials of PCBs in electronics. In fact, the printed foil laminate that is made of Cu is also called the copper-clad plate (PCB-Cu). However, PCB-Cu is inclined to suffer from surface oxidation or electrochemical corrosion. Under the polluted atmospheric environment, the dense Cu_2_O film [4] on the surface of PCB-Cu will gradually corrode and transform into Cu_4_(OH)_6_SO_4_, Cu_2_(OH)_3_Cl, Cu_2_(OH)_3_NO_3_, Cu_2_(OH)_2_CO_3_, and other corrosion products [5,6,7], losing its protective role on the substrate. Usually, certain surface treatment processes are always applied on the surface of PCB-Cu to enhance its capacity for corrosion resistance. Among these methods, electroless nickel–gold (ENIG) technology has received much attention for its outstanding corrosion resistance and high weldability. As excellent electrical contact material, corrosion cannot occur in the general atmospheric environment for a holonomic-plated layer. However, within the scope of its usual application thickness, it is easy to cause microporous corrosion [8]. The corrosion behavior of gold-plated components is closely associated with relative humidity (RH), SO_2_ [9], H_2_S [10], dust particles [11,12], molds, and other factors [13,14].

Generally, an ENIG surface treatment process can offer excellent protection for Cu. However, a multi-metal system is produced from the application of these plating metals, which aggravate the risks of electrochemical migration failure to a large extent. According to previous literature, ECM mainly consists of two failure mechanisms: the formation of dendrite [15,16,17,18] and conductive anodic filaments (CAFs) [19]. Dendritic growth usually occurs while metal ions move into the electrolyte layer near the anode and then deposit near the cathode, and growing in the formation of tree-like or needle-like. For the dendrite growth, an incubation process is pre-requisite [20]. The CAFs are formed as an anode metal coating gradually becomes ions and begins to migrate under the effects of the bias voltage [15,16,21]. The formation of dendrites and CAFs can seriously influence the reliability or security of electronic systems, especially the PCBs in electronic packages and microelectronic components. Currently, the majority of research focuses on the surface treatment process itself or the optimization of parameters, while the environmental adaptability of surface coatings needs to be further elaborated.

In this work, the ECM failure mechanisms of PCB-Cu/ENIG under thin electrolyte films of different thicknesses containing 0.1 M Na_2_SO_4_ were studied by an indoor simulation method. Moreover, a stereo microscope, scanning electron microscopy (SEM), and an X-ray energy dispersive spectrometer (EDS) were employed to investigate the surface topography and the element distribution. The scanning Kelvin probe (SKP) was used to observe the changes in surface potential of the species. At the end of the paper, the electrochemical migration corrosion failure models of PCB-Cu/ENIG are proposed.

## 2. Results and Discussion

### 2.1. Surface Topography Observation

**Figure 1:**
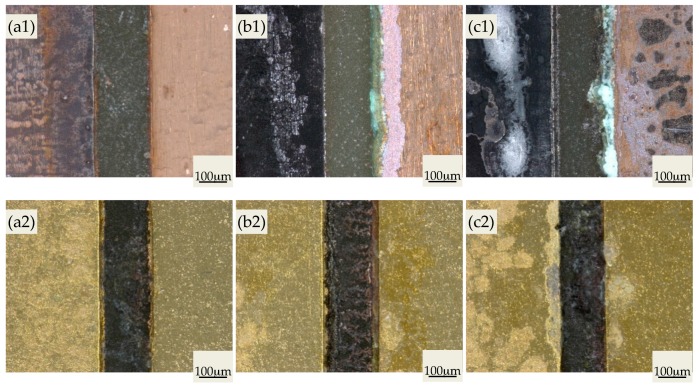
Stereo images of printed circuit boards (PCBs) after experiment under 12 V bias potential (Left: cathode plate): (**a**) 75% RH, (**b**) 85% RH, (**c**) 95% RH; (**1**) copper-clad laminate PCB (PCB-Cu) PCB-Cu, (**2**) electroless nickel/immersion gold PCB (PCB-ENIG).

Figure 1 shows the surface topographies of PCBs under experimental conditions for 24 h. Figure 1(a1) suggests that relative slight ECM corrosion occurred on PCB-Cu under low humidity (75% RH). The anode plate was smooth without any obvious changes, while the color of the cathode plate clearly turned black. Moreover, as RH increased, the color of the cathode plate continuously deepened, and the amount of salt enrichment accumulated on some localized areas of the PCB-Cu (white regions) increased. Moreover, under 95% RH, the anode plate gradually became black and rough, causing a certain degree of corrosion. Especially, the severely corrosion occurred at the anode plate edge, where there were a great number of green corrosion products that were presumably “patina”. Different from PCB-Cu, the surface of the two PCB-ENIG plates corroded little, showing only a slight degree of salt enrichment. However, the ECM behavior between the neighboring plates was more serious. Under the condition of 75% RH, significant migration of corrosion products, the deposition of metal ions, or both was found on the FR-4 board. When the humidity reached 85% RH, large amounts of red-brown tree-like analogues formed and shorted out the neighboring plates (Figure 1(b2)).

Thus, it can be concluded that, after ENIG treatment, the corrosion resistance of the PCB metal plate significantly improved, but the ECM tendency increased at the same time. On the one hand, the surface protection technique is imperfect at the side of the PCB-ENIG anode plate. Such places cannot provide effective protection on the substrate and become the center of active dissolution, providing the ion source for the ECM process. On the other hand, the electrode potential of Au is much larger than that of the intermediate Ni buffer layer and the Cu substrate. Under the synergy effects of galvanic couples and an electrical bias, the substrate metals at the micropores or defects of Au plating layer will accelerate dissolving, further aggravating the ECM process.

### 2.2. SEM and EDS Analysis

**Figure 2:**
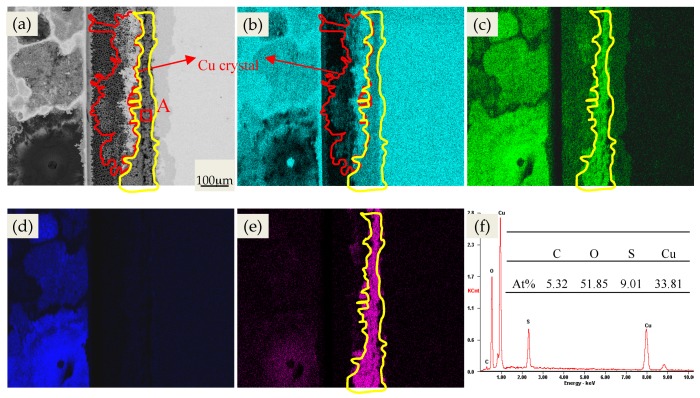
Elements distribution mapping results of PCB-Cu under the condition of 60 °C and 95% RH (Left: cathode plate): (**a**) SE; (**b**) Cu; (**c**) O; (**d**) Na; (**e**) S; (**f**) energy dispersive X-ray spectroscopy (EDS).

To observe the electrochemical migration behavior and explore the failure mechanism, elemental distribution mapping detection was performed on the PCB surface, as shown in Figure 2. From Figure 2b, it can be seen that an obvious ECM phenomenon occurred on Cu element. In the meantime, the Cu element migrated toward the cathode from the anode, and almost half of the FR-4 boards were covered. At the same time, O and S elements were also gathered at the enrichment region of the Cu element (in the yellow curve frame). In combination with EDS analysis results of Area A, the migrating corrosion products were mainly composed of sulfates and oxides/hydroxides of copper. Furthermore, it could also be found from Figure 2a that a thin layer of a bright white substance (in the red curve frame) existed on the left of the migrating corrosion products. Figure 2b shows that the bright white substance is metal Cu, indicating that a great amount of Cu ions were reduced between the two plates and that it gradually grew toward the cathode. The growth direction of the Cu crystal differs from the dendrite in previous published literature [22]. From Figure 2c,d, it can be seen that both Na and O elements were enriched on the surface of the cathode plates. Therefore, it can be deduced that the reaction on the surface of the cathode plates is mainly the O_2_ reduction reaction and the compound may be NaOH.

**Figure 3:**
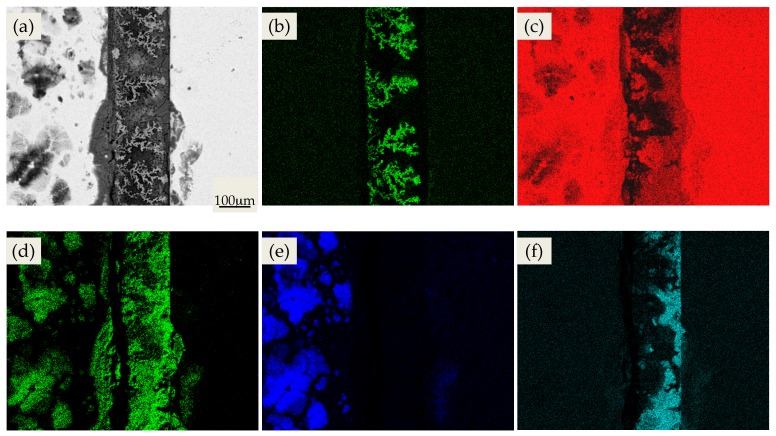
Elements distribution mapping results of PCB-ENIG under the condition of 60 °C and 95% RH (Left: cathode plate): (**a**) SE; (**b**) Cu; (**c**) Ni; (**d**) O; (**e**) Na; (**f**) S.

The element distribution mapping results of PCB-ENIG are shown in Figure 3. Figure 3b shows that Cu dendrite growth was obvious and much more serious than PCB-Cu, indicating that, under high humidity condition, Cu^2+^ from the anodic dissolution can migrate to the cathode plate and be deposited as dendrites of reverse growth. This would cause a severe short-circuit failure between the two neighboring plates for PCB-ENIG. Meanwhile, a substantial amount of Ni element also migrated. Combined with Figure 3d,f, it can be seen that the distribution of Ni element near the anode plate coincided with that of O and S elements, indicating that the migrating corrosion products of Ni were mainly composed of sulfates. Moreover, directional migration and enrichment phenomenon emerged from Na and S elements, quite similar to PCB-Cu.

### 2.3. Surface Kelvin Potentials Distribution

**Figure 4:**
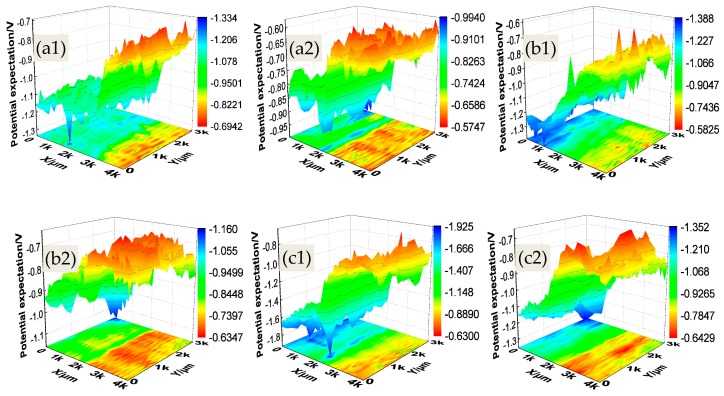
Surface Kelvin potentials distribution of PCBs after experiment (Left: cathode plate): (**a**) 75% RH, (**b**) 85% RH, (**c**) 95% RH; (**1**) PCB-Cu; (**2**) PCB-ENIG.

For the sake of observing the changes of surface potential of both anode plates and cathode and elaborate the corrosion behavior and rules of PCBs, the surface Kelvin potentials *E*_kp_ of PCBs under different humidity conditions were measured, shown in Figure 4. The principles of SKP techniques are mainly to achieve the surface potential difference of species by the measurement of the electronic work function of metal surface. The previous studies suggested that the potential *E*_kp_ has a linear dependence with the corrosion potential *E*_corr_ in air (Equation (1)) [23,24,25]:

(1) $$E_{\mathrm{corr}}=\left(\frac{W_{\mathrm{ref}}}{F}-\frac{E_{\mathrm{ref}}}{2}\right)+E_{\mathrm{kp}}$$

wherein *W*_ref_ is electrode work function; *E*_ref_/2 is the half-cell potential of the Kelvin probe. Under a specific system, these two parameters are constant. Therefore, the changes of *E*_kp_ reflect the variation in the corrosion potential.

**Figure 5:**
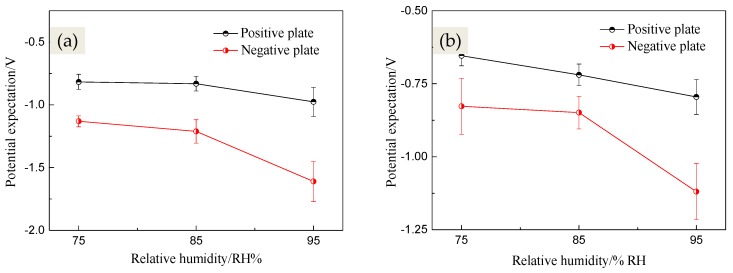
Curves of E_kp_ expectations vs. RH for PCB-Cu (**a**) and PCB-ENIG (**b**).

Gauss fitting were conducted for the surface Kelvin potentials distribution of both cathode and anode plates, respectively. Equation (2) shows the fitting formula:

(2) $$y=y_{0}+\frac{A}{w\sqrt{2\pi }}\exp\left(-\frac{{\left(x-\mu \right)}^{2}}{2w^{2}}\right)$$

wherein *μ* is the potentials expectation value that is the centralized location of potentials distribution; *σ* = *w*/2 is the standard deviation of the Gaussian distribution, which represents the dispersion degree of the potentials distribution. The greater the value *σ* is, the more dispersed the potentials distribution is. Figure 5 shows the Gauss fitting results (potential expectation) for the XY walls of 3D potential mapping.

Figure 4 and Figure 5 show that the surface Kelvin potentials distribution of PCB-Cu plates under different RH condition had a similar rule: the potential of the cathode plate was markedly lower than that of the anode plate. This suggests that the surface states of PCB-Cu changed significantly under the effect of the bias voltage, and these changes would not vanish, even after eliminating the bias voltage. With the RH increasing, the potentials of both anode and cathode plates exhibited a descending trend, but the potentials of the cathode plate fell even more sharply. Consequently, the potential difference between anode and cathode continuously increased and reached a maximum value of 0.635 V under 95% RH. This phenomenon can be explained as follows: for the anode plate, a large amount of metal cation was produced due to corrosion under the bias voltage, and these cation then migrated toward the cathode. Meanwhile, the reduction reaction of oxygen (O_2_ + 2H_2_O + 4e^−^ → 4OH^−^) occurred on the cathode, and the hydroxide ions migrated toward the anode. However, the migration ability of hydroxide ion is more superior due to the smaller ionic radius and the migration resistance [15,26]; thus, the corrosion products piled on the anode. It was noted that the accumulation of corrosion products on the surface of the anode plate hindered the surface electron emitting process to some extent and resulted in much nobler potentials mapping. Moreover, the thickness of the electrolyte layer gradually increased with the increase in relative humidity, which caused a decrease in resistance ion migration. Under this condition, it became much easier for the cation to migrate, which led to a smaller amount of corrosion products accumulating on the anode. Consequently, the potential of the anode plate decreased as relative humidity increased. For the cathode plate, the bias voltage caused a cathodic protection effect. When the relative humidity increased, the cathode current density augmented, which exhibited a better protection effect. Therefore, the corrosion extent of the cathode was alleviated and the potential of it also reduced.

The evolution law of the surface Kelvin potentials distribution of PCB-ENIG under different RH conditions was the same as that of PCB-Cu. However, the potential expectations of PCB-ENIG were higher than that of PCB-Cu overall, suggesting that the ENIG treatment could effectively reduce the corrosion tendency of PCB-Cu. Meanwhile, the potential difference between the anode and cathode plates of PCB-ENIG was much smaller compared to PCB-Cu. The maximum potential difference, 0.324 V, for PCB-ENIG, was quite close to the minimum potential difference of PCB-Cu under 75% RH condition, 0.314 V.

### 2.4. ECM Failure Model

ECM under the thin electrolyte film is the major corrosion failure mechanism of PCBs in the test conditions. For PCB-Cu, Equations (3)–(5) are the main anodic reactions:

(3) $$\mathrm{Cu}\to {\mathrm{Cu}}^{+}{+e}^{-}$$

(4) $${\mathrm{Cu}}^{+}\to {\mathrm{Cu}}^{2+}{+e}^{-}$$

(5) $$\mathrm{Cu}\to {\mathrm{Cu}}^{2+}{+2e}^{-}.$$

The bias voltage applied in this experiment is relative large (12 V); therefore, the metal ions from the dissolving PCB-Cu anode plate should be mainly Cu^2+^. Furthermore, some H_2_O was decomposed under such a potential value, producing a small amount of H^+^ [27]. Equation (6) shows the decomposition reaction:

(6) $${2H}_{2}O\to O_{2}{+4H}^{+}{+4e}^{-}.$$

The main reaction occurring on the cathode plate was the reduction of O_2_, as shown in Equation (7):

(7) $$O_{2}{+2H}_{2}O\to {4\mathrm{OH}}^{-}-{4e}^{-}.$$

**Figure 6:**
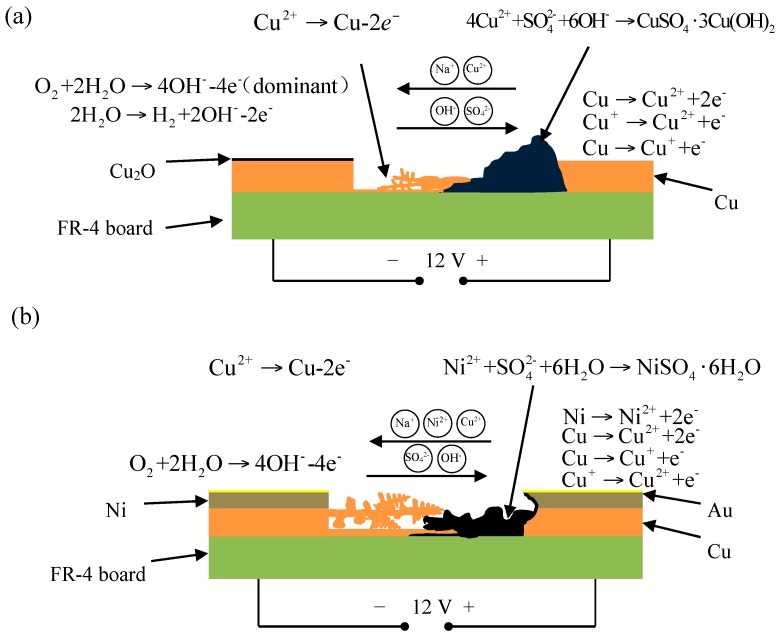
ECM corrosion model of PCB-Cu (**a**) and PCB-ENIG (**b**).

As can be seen from Figure 6a, under the effects of the bias voltage, both anions and cations migrate directionally and this phenomenon is further aggravated with the increase in RH, which led to the enrichment phenomenon of some specific ions (for example, Na^+^ and SO_4_^2−^) between two different plates. Overall, PCB-Cu has a poor corrosion resistance and dissolved rapidly under the synergy effects of electrolyte ions and a bias voltage. When the RH is low, the resistance of the ion migration is very great due to the extremely thin electrolyte layer. Under this condition, the ECM is quite slight. The resistance of ion migration gradually decreases as relative humidity increases. In the meantime, the cation (Cu^2+^) and cathodic ion (OH^−^, SO_4_^2−^) migrate in the opposite direction. Moreover, a part of the cation encounters the cathodic ion in the vicinity of the anode plate and causes an accumulation of corrosion products (Equation (8)) due to the much faster migration rate of OH^−^. In addition, the other part of Cu^2+^ was reduced as Cu formed, and stretched toward the cathode (Figure 2). It was noted that the reduced distance between the two plates due to Cu crystal formation would lead to a strong gradient of electric field to the Cu tip, consequently making subsequent Cu^2+^ deposition to continue at this site. However, as the amount of Cu^2+^ decreased, at that moment, the Cu dendrite turned into faint filamentary morphology (Figure 2a).

(8) $${4\mathrm{Cu}}^{2+}{+\mathrm{SO}}_{4}^{2-}+6{\mathrm{OH}}^{-}\to {\mathrm{CuSO}}_{4}\cdot {3\mathrm{Cu}(\mathrm{OH})}_{2}(\mathrm{or}\;{\mathrm{Cu}}_{4}({\mathrm{SO}}_{4}){\left(\mathrm{OH}\right)}_{6}).$$

The model of ECM failure for PCB-ENIG is shown in Figure 6b. Similar to PCB-Cu, directional migration of some specific ions occurred. The difference was that the Ni intermediate layer was also involved in the electrochemical migration processes, in the form of Ni^2+^. As to the Au plating layer, the dissolving and migrating process can be ignored due to its excellent corrosion resistance and high electrode potential [28]. At the initial stage of the ECM process, the Ni intermediate layer within the micropores or defects [29] of Au plating layer first dissolves and migrates out as Ni^2+^ from the side of the anode plate. When Ni^2+^ meet SO_4_^2−^, Ni_2_SO_4_·6H_2_O will precipitate out (Equation (9)), accompanied by the formation of Ni_4_(OH)_2_(SO_4_)_3_·*y*H_2_O (Equations (10) and (11)) [1].

(9) $${\mathrm{Ni}}^{2+}{+\mathrm{SO}}_{4}^{2-}{+6H}_{2}O\to {\mathrm{NiSO}}_{4}\cdot {6H}_{2}O$$

(10) $${\mathrm{Ni}}^{2+}{+2\mathrm{OH}}^{-}\to {\mathrm{Ni}(\mathrm{OH})}_{2}$$

(11) $${\mathrm{Ni}(\mathrm{OH})}_{2}{+3\mathrm{Ni}}^{2+}+3{\mathrm{SO}}_{4}^{2-}+6yH_{2}O\to {\mathrm{Ni}}_{4}{\left(\mathrm{OH}\right)}_{2}{{(\mathrm{SO}}_{4})}_{3}\cdot yH_{2}O.$$

Along with the dissolution of the Ni intermediate layer, the Cu substrate will be gradually exposed, and the ECM process will begin. However, the precipitating of Ni_2_SO_4_·6H_2_O and Ni(OH)_2_ reduces the concentration of SO_4_^2−^ and OH^-^ near the anode plate. As a result, there are few corrosion products containing copper deposited in the vicinity of the anode plate (Figure 3b). Moreover, under the effects of an electrical bias, Cu^2+^ can smoothly migrate to the cathode plate and plate out, forming Cu dendrites of reverse growth. This increases the ECM tendency of PCB-ENIG sharply, and obvious Cu dendrite growth can be observed under experimental conditions (Figure 3b).

## 3. Materials and Methods

### 3.1. Material Preparation

**Figure 7:**
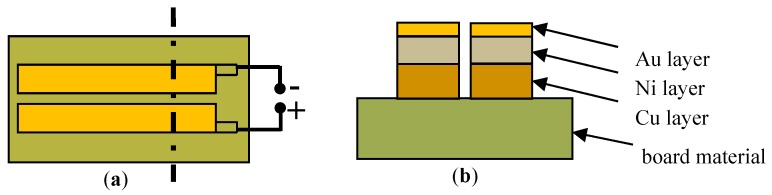
Schematic diagram of the PCB-ENIG sample: (**a**) vertical diagram and (**b**) cross-sectional diagram.

PCB-Cu and PCB-ENIG produced by Sprine Co. in China were elected as experimental materials, as shown in Figure 7. Table 1 showed the basic parameters of PCBs. The effective size of printed metal plates is 3 mm × 30 mm, and the spacing between two neighboring plates is 0.2 mm. Prior to experiment, all samples were degreased with acetone for 5 min, washed with distilled water for 3 min, and dried by a compressed cold air flow.

### 3.2. Experimental Method

To simulate the ECM corrosion behavior of PCB-Cu/ENIG under the atmospheric environment with industrial pollution, the electrolyte solution is sprayed on the surface of all samples in the same manner (the same height, the same number of spraying and perpendicular to the sample). Thus, it assumes that the thickness of the electrolyte layer distributed on the specimen is the same and uniform. Then, the specimens were placed into a constant temperature and humidity test chamber. After absorbed thin electrolyte layers of different thicknesses were produced on the PCBs, a 12 V bias voltage was applied between the two neighboring plates. During the experiments, the temperature was constant at 60 °C, and the relative humidity was 75% RH, 85% RH, and 95% RH, respectively. After 24 h tests, the bias voltage was removed, and all specimens were taken out to surface topography (dendrite) observation and SKP tests.

The surface topography and the dendrite growth behavior of PCB-Cu/ENIG were observed using 3D stereology macroscopy (VHX-2000, Keyence, Japan) and an environment scanning electron microscope (ESEM, FEI Quanta 250, USA) with an electron-accelerated voltage of 20 keV; and the distribution of elements was analyzed by energy dispersive X-ray spectroscopy (EDS). An M370 scanning Kelvin probe was employed to observe the volta potentials of the sample surfaces. The work distance was 100 ± 2 μm between the tip and samples. The size of scanning area was 4000 μm × 3000 μm. The step size at the *x*-axis was 100 μm, while it was 200 μm at the *y*-axis. These experiments were conducted in a stable laboratory environment of 25 °C and 60% RH.

## 4. Conclusions

The ECM behavior and mechanism of PCB-Cu and PCB-ENIG under absorbed thin electrolyte films with different thicknesses were investigated in this work. When applied 12 V bias voltage, for PCB-Cu, copper dendrites and sulfate precipitates were found on the FR-4 board between two plates. Moreover, the Cu dendrite was produced between the two plates and migrated toward the cathode. Compared to PCB-Cu, PCB-ENIG had a high electrochemical migration tendency and a serious short-circuit failure would occur under a high RH environment. Furthermore, the surface potential of the cathode plate became lower than that of the anode plate after bias application, and the potentials of the anode and cathode plates exhibited a descending trend as the relative humidity increased.

## Figures and Tables

**Table 1:** The parameters and treatments of PCB samples.

Items	Board 1	Board 2
Board Materials	FR-4	FR-4
Board Thickness (mm)	0.8	0.8
Thickness of Copper Foil (μm)	25–30	25–30
Surface Treatment	no	ENIG
Thickness of Protective Layer (μm)	0	0.02
